# Advancements in Hysteroscopic Diagnosis and Management of Endometritis

**DOI:** 10.3390/diagnostics15030243

**Published:** 2025-01-21

**Authors:** Alkis Matsas, Dimitrios Stefanoudakis, Georgia Kotsira, Sofoklis Stavros, Spyridon Gkoufas, Nikoletta Vrettou, Smaragdi Christopoulou, Panagiotis Christopoulos

**Affiliations:** 1 Second Department of Obstetrics and Gynecology, Medical School, “Aretaieion” University Hospital, National and Kapodistrian University of Athens, 11527 Athens, Greece; amatsas@med.uoa.gr (A.M.);; 2 Third Department of Obstetrics and Gynecology, Attikon Hospital, Medical School, National and Kapodistrian University of Athens, 12462 Athens, Greece

**Keywords:** chronic endometritis, hysteroscopy, infertility, diagnosis, treatment, antibiotic therapy, reproductive outcomes, fertility benefits

## Abstract

Infertility remains a complex clinical challenge, with intrauterine pathologies contributing to a significant percentage of in vitro fertilization (IVF) failures. Chronic endometritis (CE) has gained attention due to its potential association with unexplained infertility and recurrent miscarriage. This review explores the role of hysteroscopy in diagnosing and treating CE. The endometrium undergoes dynamic changes orchestrated by ovarian steroids, and disturbances may lead to CE, characterized by plasma cell infiltration. Diagnosis traditionally relies on histopathologic examination, but hysteroscopy offers real-time imaging, revealing the specific macroscopic alterations associated with CE. However, diagnostic accuracy varies, prompting the need for standardized criteria. CE has been linked to poor reproductive outcomes, emphasizing the importance of effective treatment. Antibiotic therapy is a common approach, with doxycycline as the first-line regimen. Hysteroscopic polypectomy, targeting non-infectious CE, emerges as a promising treatment, demonstrating fertility benefits. The review underscores the significance of hysteroscopy in diagnosing and treating CE, providing insights into its impact on reproductive outcomes in infertile women. Further prospective studies are needed to validate these findings and establish unified diagnostic criteria.

## 1. Introduction

Burgeoning scientific interest has been concentrated on chronic endometritis and its reproductive sequelae. Multiple factors, including embryo, uterine, and technical issues, may contribute to the failure of in vitro fertilization cycles. It has been acknowledged that intrauterine pathologies account for 10–15% of IVF failures [1]. The number of publications focused on chronic endometritis and infertility has increased significantly due to the emergence of data suggesting the possible involvement of chronic endometritis in reproductive outcomes, indicating the increase in its prevalence in women with unexplained infertility and recurrent miscarriage [2].

The endometrium is a distinct mucosal tissue inside the uterine corpus that undergoes dynamic morphologic, transcriptional, and translational changes on a monthly basis, orchestrated by the two principal ovarian steroids, 17beta-estradiol and progesterone [3]. Numerous immunocompetent cells, including natural killer (NK) cells, macrophages, neutrophils, dendritic cells, and various subsets of T cells, penetrate the human endometrium during the menstrual cycle. Within a menstrual cycle, these endometrial leukocyte subpopulations’ composition and density are additionally altered regularly. It is believed that these timely changes in the mucosal leukocyte subpopulations constitute a prerequisite for the establishment of embryo implantation and placentation [4].

Endometritis is an inflammatory disease of the uterus characterized by the presence of plasma cells in the endometrium. It is classified into the following two types: acute and chronic. Acute endometritis is a symptomatic acute inflammation of the endometrium characterized by micro-abscess and neutrophil invasion in the superficial endometrium when examined under a microscope [5]. Acute endometritis can be identified by systematic fever, pelvic pain, and increased vaginal discharge [6]. In striking contrast to acute endometritis, chronic endometritis is a silent disease that is usually discovered during a secondary amenorrhea and infertility workup. Tuberculosis is a major cause of chronic endometritis, particularly in developing countries. Chronic and acute endometritis has been linked to poor reproductive outcomes [5].

According to the bibliography, due to a lack of distinctive symptoms, diagnosis of chronic endometritis (CE) is quite challenging [7]. CE is a benign gynecologic condition that affects nearly 10% of women and is characterized by abnormal uterine bleeding (AUB) or pelvic pain. Although the vast majority of patients experience pain, due to the lack of histologic or microscopic confirmation of CE involvement, the disease remains largely asymptomatic [7]. CE is a disease of continuous and subtle inflammation characterized by plasma cell infiltration into the endometrial stromal area, where they are normally absent except just before and during menstruation [8]. CE has traditionally not been a concern in clinical practice because it is usually asymptomatic or only manifests with subtle symptoms such as abnormal uterine bleeding, pelvic pain, dyspareunia, and leucorrhea [9]. Thus, the asymptomatic or oligosymptomatic nature of CE has hindered basic and clinical research on this disease [6].

Taking into account all the above, CE is difficult to identify even though histological examinations can be used to establish the diagnosis. There is no specific sign for either pelvic examination or transvaginal sonography. Fluid hysteroscopy, however, is a dependable method for detecting CE [10]. Fluid hysteroscopy, which uses saline to ensure smooth distension of the uterine cavity and floating of new growths, allows for more subtle detail of the uterine cavity [10]. The role of hysteroscopy in various benign gynecologic conditions is well documented, but in the case of CE, the scientific community is divided on whether hysteroscopy is appropriate for detecting cases of chronic or subclinical endometritis [11].

## 2. Methods

**Figure 1:**
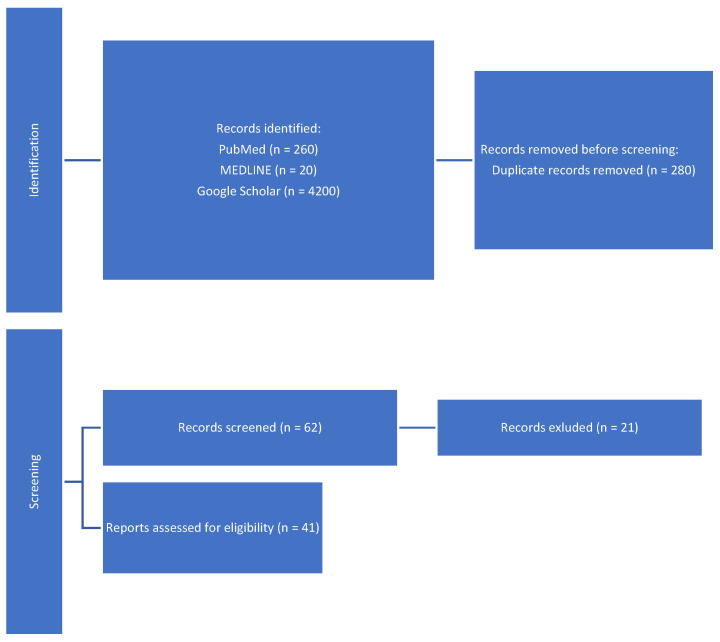
Selection of papers.

A literature search was performed on the following databases: MEDLINE, Google Scholar, Scopus, and PubMed database. We searched these databases for studies published until February 2023 in the English language in order to elucidate the role of hysteroscopy in the diagnosis and treatment of CE. The literature search was conducted using the combination of the following Medical Subject headings (MeSH) and relevant keywords in different orders: “endometritis”, “acute”, “chronic”, “management”, “diagnosis”, “immunohistochemical”, “hysteroscopy”, “infertility”, “pathophysiology”, and “reproductive outcome”. The reference lists of the included studies were also checked to look for studies that were not found in the electronic literature search. Original articles and some review articles published within the last five years were given priority. All articles were accessed in full text (Figure 1). In this review, individual data sources were not sought for, and a descriptive analysis was carried out. The data were then summarized in the form of a descriptive review.

## 3. Results

Until recently, the human uterine cavity was thought to be aseptic when common microscopic and culture-based techniques were used to discover bacterial colonies. However, high-throughput methods using 16s rRNA gene sequence analysis have demonstrated the existence of bacteria in the uterine cavity [6]. Bacterial communities in the human body have been vital to our health, but their imbalance, malfunction, and maldistribution (dysbiosis) can lead to numerous kinds of diseases [6]. Thus, bacteria from the vagina and cervix, which migrate into the uterus, alter the uterine and endometrial microbiota and this consequently leads to endometritis. When the endometrium undergoes devitalization and damage (as in the case of a caesarean section or uterine surgery), it tends to harbor microorganisms. Any pelvic procedure that involves dilatation, curettage, or endometrial aspiration increases the risk of endometritis if proper asepsis is not maintained or if the patient has an untreated vaginal infection. Patients without risk factors might still develop endometritis after a typical spontaneous vaginal delivery, with an incidence of 1% to 2%. Patient incidence rises to 5–6% in those with risk factors. Risk factors include being a young woman from a lower socioeconomic background, having a high BMI, experiencing a prolonged rupture of the membranes, having multiple per vaginal exams, monitoring or sampling of the fetal scalp, having chorioamnionitis, having meconium-stained amniotic fluid, and having an undiagnosed untreated vaginal infection [5].

As mentioned above, traditional chronic endometritis screening methods focus on histology, which is based on the detection of plasma cells in the endometrial stroma, but this method is vague and reliant on the timing of the menstrual cycle when sampling occurs [12]. Considering these limitations, fluid hysteroscopy is a helpful tool that allows for the real-time imaging of the uterine cavity and is widely employed in gynecologic practice for diagnosis and treatment of chronic endometritis [6].

### 3.1. Diagnosis

CE is characterized by a prolonged, mild endometrial inflammation, as well as by plasma cell infiltration into the endometrial stromal area. Because chronic endometritis is difficult to diagnose, its prevalence is frequently underestimated. In particular, the prevalence of chronic endometritis ranges from 0.2% to 46%, depending on patient profile and biopsy method [5]. CE affects the lining of the endometrium and is defined by the infiltration of massive plasmacytes in the endometrial stromal tissues, known as endometrial stromal plasmacyte (ESPC), high stromal cell density, and dissociated maturation between the epithelium and stroma. The pathogenesis of CE appears to be associated with a qualitative and quantitative change in the endometrial microbiome, with abnormal proliferation of various microorganisms, primarily Gram-negative and intracellular bacteria (such as *Enterococcus faecalis*, *Mycoplasma*, *Ureaplasma*, *Chlamydia*, *Escherichia coli*, and *Streptococcus* spp.) [13]. CE develops in women of childbearing age as a result of sexual activity, improper uterine cavity operation, pathogenic microorganism invasion, and other factors. CE patients typically have no or mild symptoms. Symptoms range from none at all to pelvic pain, vaginal discharge, dyspareunia, and abnormal vaginal bleeding, and approximately 25% of patients with CE are asymptomatic. Furthermore, the existence of CE is not predicted by peripheral blood inflammation markers such C-reactive protein (CRP), leukocytosis, leptin, and IL6 [14]. Thus, the diagnosis of CE is a challenge for gynecologists. For the diagnosis of CE, the following two methods are commonly used: hysteroscopy and the identification of plasma cells in endometrial biopsy. The classic diagnosis of CE is based on histopathologic examination and endometrial biopsy, with the presence of endometrial stromal plasma cells serving as the primary diagnostic marker [15].

The histological identification of plasma cells in the stromal region of the endometrium in all extracted patient endometrial tissues is the gold standard for the diagnosis of CE. Besides the presence of plasma cells, other relevant indications include strong stromal cell proliferation, segregated maturation between the epithelium and stroma, and significant pre-decidual reaction. In general, plasma cells are larger and have an eccentric nucleus surrounded by abundant basophilic cytoplasm [16]. The detection of plasma cells can be confirmed with strains such as hematoxylin and eosin (HE). Nevertheless, applying conventional staining methods might sometimes make it impossible to identify plasma cells in microscopic examination due to the existence of mononuclear cell infiltration, mitosis and proliferation of stromal cells, plasmacytoid presence of stromal cells (fibroblasts and mononuclear cells), or decidual transformation of the endometrium during the late secretory phase [14].

In order to improve the accuracy of microscopic examinations and lower the possibility of misdiagnosis, the inclusion of immunohistochemical staining for syndecan-1 has proven to be mandatory. Syndecan 1 is a transmembrane heparan sulfate proteoglycan found on the surface of plasma cells and keratinocytes but not on mononuclear cells, lymphocytes, or endometrial stromal cells. It is additionally known as CD138, and it aids in the detection of plasma cells and the presence of CE while remaining unaffected by intra- and interobserver variability [14]. As CD138, is a plasma cell marker which stains well on plasma cell surfaces, CE is clinically diagnosed via immunohistochemistry (IHC) [16]. When ≥5 typical plasma cells were observed in the endometrial stroma at a magnification of 400, the CD138 immunohistochemical diagnostic criteria identified CE as the condition. No CE was identified when <5 or no typical plasma cells were detected in the endometrium [17].

**Figure 2:**
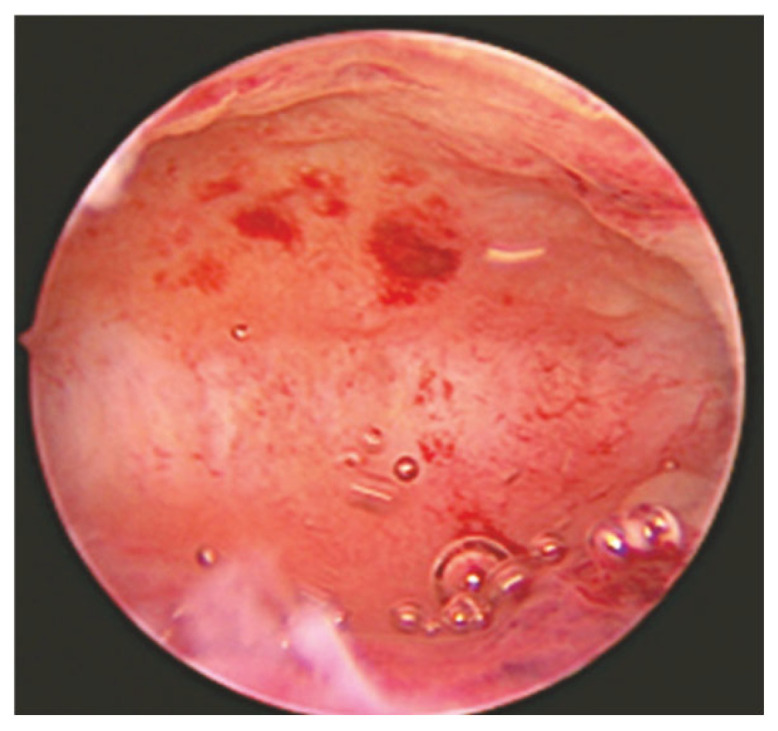
Focal endometrial hyperemia: Foci and hyperemia spots are shown in the endometrium [23].

**Figure 3:**
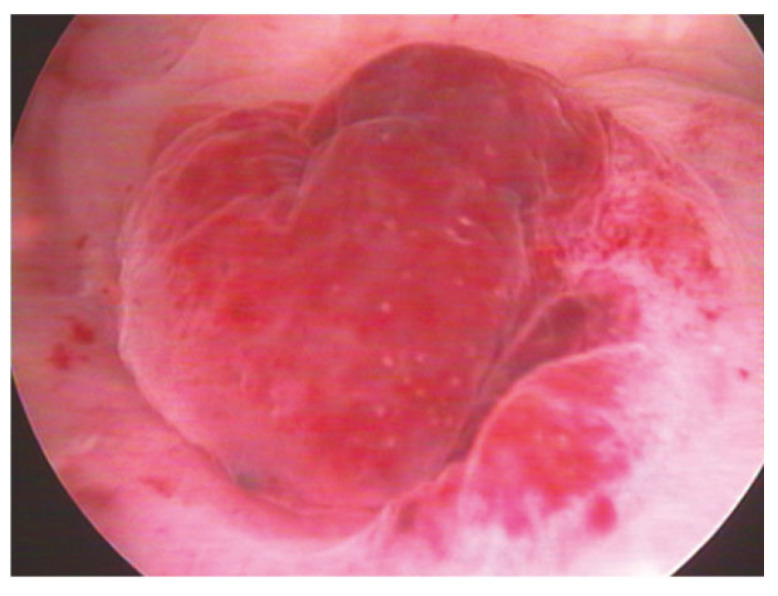
Hyperemic polyp and multiple red spots: endometrium shows irregular thickness [23].

**Figure 4:**
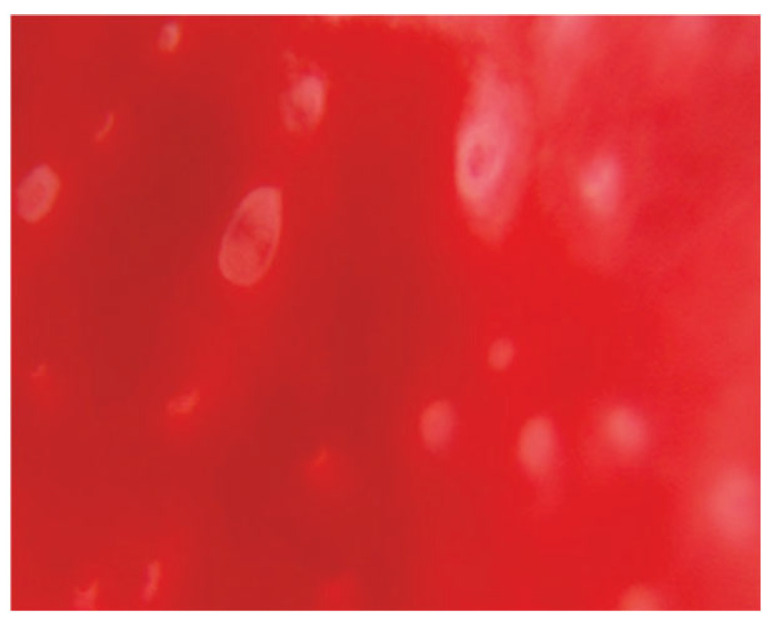
Strawberry appearance and hyperemia at close observation [23].

Even though the role of hysteroscopy in a number of benign gynecological disorders is well established, the scientific community is divided on whether hysteroscopy is effective in identifying cases of chronic or subclinical endometritis in CE cases. Every time hysteroscopy is performed to evaluate the endometrial cavity, there is a significant heterogeneity between the findings and the techniques employed to demonstrate the endometritis, according to the literature [18]. Based on recent studies, specific macroscopic alterations of the endometrium that are associated with CE, such as mucosal edema and micro-polyps, can be detected via hysteroscopy. In particular, the commonly described hysteroscopic patterns that are suggestive of CE include micro-polyps (small pedunculated, vascularized protrusions of the uterine mucosa measuring <1 mm), focal or diffuse endometrial hyperemia (Figure 2) (accentuated blood vessel accumulation at the peri-glandular level) and mucosal edema (pale and thickened endometrium in the proliferative phase) (Figure 3), [16]. When the uterine cavity is directly examined, areas of red endometrium flushed with a white central point can be detected as localized or dispersed throughout the cavity, realizing a characteristic feature known as the “strawberry aspect” [14] (Figure 4). Thus, the term “strawberry effect” includes focal or diffuse hyperemia, stromal edema, and homogenous or non-homogenous endometrial thickening [18].

Additionally, these findings were confirmed in a more recent study conducted by Vaduva et al. in an attempt to correlate infertility with endometritis and to determine the success rate of IVF after endometritis has been diagnosed and treated. A total of 446 couples were investigated by hysteroscopy, the only investigation that allows for the direct visualization of the uterine cavity. Similar lesions were identified in 52% of the cases, which also matched the diagnostic patterns we described above. In more detail, hypervascularity, congestion, mosaic, and red-dotted plaques with vascular dystrophy were more prevalent. Notably, at the peri-glandular region, the vascular network was characterized by vascular dystrophy and localized or diffuse peri-glandular hyperemia. There were focal or sparsely distributed bright red endometrial regions with central white spots that gave the endometrium surface a “strawberry-like” appearance. In direct contact, these regions bleed effortlessly. Endometrial hypertrophy, whether localized or diffuse, was also common. Another distinct symptom of CE that we found through hysteroscopy was scattered or localized polyposis. Micro-polyps were often present, and they were simple to find since they floated in the distension liquid [19].

The diagnostic accuracy of hysteroscopy varies significantly across studies, considering the lack of agreement on the endometrial characteristics of CE (sensitivity and specificity range from 40% to 100% and from 56% to 92.5%, respectively, with the use of immunohistochemistry for CD-138 as a reference standard) [15]. For instance, when edema and hyperemia detection was selected as a criterion of CE in the study by Cicinelli et al., 92% sensitivity, 93% specificity, 64% positive, and 99% negative predictive values were reported [20,21]. However, when micro-polyps were additionally taken into account, the specificity rose to 99.0% while the sensitivity fell to 55.4% [15]. The authors came to the conclusion that the absence of endometrial hyperemia and edema was enough to rule out chronic endometritis, while the presence of micro-polyps was a very trustworthy visual sign, although uncommon in CE patients [20].

It is important to note that these findings may be biased because the histopathological verification method did not employ ICH staining [21]. Moreover, Cicinelli et al. used CD-138 immunoreactivity, which is specific for inflammation, to suggest the positive for CD-138 in terms of histologic evidence of CE. Endometrial polyps were not always associated with CE, therefore the shared pattern of inflammation in both endometrial polyps and CE only suggests a potential relationship between the two [15]. The wide range of hysteroscopy diagnostic accuracy reported by several research groups resulted in the necessity to establish a diagnostic agreement. Using the Delphic technique, the “Working Group for Standardization of Chronic Endometritis Diagnosis” came to an agreement in 2019. The existence of at least one of the following hysteroscopic features complied with the experts’ established diagnostic standards: strawberry appearance, focal hyperemia, hemorrhagic patches, micro-polyps, and stromal edema in the follicular phase [15,22].

The primary drawback of a hysteroscopic examination is that the visual evaluation of the uterine cavity is subjective and may be influenced by the physician’s level of experience [22]. Liu et al., with the intention to eliminate the interobserver variability and based on the above-described hysteroscopic suggestive patterns, created a hysteroscopic scoring system with high sensitivity and specificity for CE assessed by ROC analysis. In particular, the principal factors evaluated by logistic regression and scored. Endometrial diffuse hyperemia scored four, hemorrhagic spots scored two, focal hyperemia scored two, dilated endometrial vessels scored two, micro-polyps scored one, polyps scored one, and history of repeat artificial insemination failure scored two. The scoring system had a maximum score of 14 and a minimum score of 0. According to the ROC curve and the maximum value of the Youden Index, the cutoff value was >2, excluding 2. A score of >2 indicated high predictability for the CE diagnosis and poor predictability for the other diagnoses [17] (Table 1).

There are two main approaches to perform a hysteroscopy, which are through the vagina or by testing the vaginal speculum. Applying thin endoscopes (less than 4 mm) is proposed to patients that have an inflamed uterus and are at risk of feeing discomfort compared to other women. Lens-based mini-hysteroscopes (2.7 mm OD mini-telescope), equipped with a 3.5 mm OD single-flow diagnostic covering are used. Generally, endometrial biopsies should be executed in the follicular phase. Moreover, saline with a pressure of around 50–60 mmHg should be chosen over CO_2_ to distend the uterine cavity. In comparison to CO_2_, saline provides a gentler dilation of the endometrium. During hysteroscopy, both the anterior and posterior walls of the endometrium must be examined. This can be performed by carefully inspecting the surface of the endometrium and identifying any irregularities [23].

Endometrial biopsy and histopathologic examinations in combination with immunohistochemistry for CD138 (IHC- CD138) have always been the gold standard method for the diagnosis of CE. However, the prediction of histopathologic CE based on IHC-CD138 derived from hysteroscopic CE data is moderate (approximately 60–70%) [24,25,26] and also relies on the gynecologists’ subjective diagnostic analysis. In order to reduce misdiagnoses, artificial intelligence (AI) plays a crucial role in minimizing human errors. Mihara M et al. [27] proposed that artificial intelligence significantly contributes to improvements in the chronic endometritis’ diagnostic process by increasing the precision of medical image analysis and combining several diagnostic methods to offer an accurate evaluation. One promising diagnostic tool for IHC is dual immunohistochemistry for CD138, while the multiple myeloma oncogene 1 (MUM-1) has also been suggested as a possible marker, with the main disadvantage of this method being that the lower limit of plasma cells per tissue sample has not been established in order to safely diagnose CE [27]. Therefore, the main challenge is to establish definite criteria for the diagnosis of CE, as it is up to each scientist to decide which method to use. Initially, AI algorithms are applied in immunohistochemistry analysis to identify CD-138+ and MUM-1+ ESPCs that are indicative for CE in images with tissue staining. This automatic identification leads to more accurate diagnoses and reduces the false-positive results as it is capable of promptly predicting the placement of these cells. Furthermore, computer-aided diagnosis (CADx) helps in the evaluation of hysteroscopic images, while pinpointing areas that may require additional investigation. For instance, Visual Geometry Group 16 (VGG-16), which is a deepConvolutional Neural Network (CNN) architecture that has found significant applications incomputer vision, was used in the examination of endometrial micro-polyposis. This model established high diagnostic precision and can be comparable with a skilled gynecologist’s diagnostic insight. Additionally, submucosal fibroids, endometrial polyps, endometrial cancer and atypical hyperplasia can be differentiated by CADx. Thus, this model can aid in the automatic classification of endometrial abnormalities. K. Kitaya et.al. [28] planned to create a convolutional neural network (CNN) model by using archival fluid hysteroscopy (F-HSC) images for the automatic detection of endometrial micro-polyps (EMiP), an F-HSC finding recognized as tiny protruding lesions strongly associated to CE. Convolutional neural network (CNN) is currently one the most developed deep learning models. In contrast to doctors who typically provide one decision (presence or absence) [29], CNN models are capable of forecasting the probability of the disease. F-HSC is becoming more prevalent as a diagnostic tool due to its ability to examine the whole uterine cavity and compared to endometrial biopsy is less invasive.

### 3.2. Treatment

Considering that CE is typically asymptomatic or only manifests as mild symptoms such abnormal uterine bleeding, pelvic pain, dyspareunia, and leucorrhea, it has not traditionally been a concern in clinical practice. As a result, it was considered as a benign condition for which the aim of diagnosis and treatment were unclear while endometrial specimens remain necessary, which can be unpleasant for the patient. This point of view has allowed the specialty of gynecology to understate the significance of chronic endometritis. Recent studies, however, have revealed that CE has a negative impact on fertility, suggesting a pathological role for CE and the need for effective treatment tools [16].

An adequately functioning endometrium is a necessary factor for successful implantation and the development of pregnancy [30]. Leukocytes, cytokines, chemokines, and other endometrial factors are among the physiological compounds of the implantation process. The regulation of the immune response and the growth of the trophoblast are both crucially regulated by all of these cells and their mediators The presence of CE can change the endometrium’s receptivity, resulting in an inadequate microenvironment that prevents typical implantation [14].

Up to 56.8% of infertile women have chronic endometritis, which is twice as likely as in these women as it is in fertile ones and more prevalent than it is in the general population [14,31,32]. This frequency is significantly higher in women with RIF and reoccurring miscarriage, according to estimates of up to 67.5% [33] and up to 67.6% [34]. According to Volodarsky Perel et al., women with endometrial polyps who have an undiagnosed history of infertility have a significantly higher prevalence of chronic endometritis than women without such a history (22.6% versus 8.6%, *p* = 0.001) and the risk of future infertility is 60% higher in women of reproductive age who have chronic endometritis than in women without the condition [35].

The need for a treatment which is not solely for symptoms but rather directed at long-term fertility-related benefits is therefore widely accepted. The main treatment option is the appropriate administration of antibiotic therapy; however, hysteroscopic polypectomy also appears to have significant fertility benefits. According to Vaduva et al., the main cause of CE is an endometrial infection brought on by bacteria that regularly inhabit the lower vaginal tract. Thus, the most usual implicated agents, which can be identified with microbiological cultures, are pyogenic pathogens such *Streptococci*, *Staphylococci*, *Enterococci*, and *E. coli*. But there are also cases of bacteria such *Chlamydia trachomatis*, *Mycoplasma*, and *Ureaplasma* [19]. Worth mentioning is that only microorganisms capable of growing under standard microbiology laboratory settings can be recovered, and the technique might consequently produce biased microbiological results. As a result, negative bacterial culture results are therefore more likely to be the result of technique-related problems than a lack of microbes in the uterine cavity [21]. Thus, it cannot be ruled out that other microorganisms (anaerobic bacteria, viruses, etc.) may coexist and play a role [31]. The detection of low biomass uterine microbiota appears to be possible by the use of molecular methods. Quantitative polymerase chain reaction and next-generation sequencing of the 16S RNA bacterium are two examples of these methods [21].

Depending on the results of the endometrial aspiration/biopsy’s culture and Gram stain, oral antibiotics are the gold standard for the treatment for chronic endometritis; a biopsy of the endometrium is repeated after the prescribed period of treatment [5]. As mentioned above, because culture might not be an accurate method for identifying the microorganisms implicated, broad-spectrum antibiotics have been proposed as recommended therapy [2]. Consequently, chronic endometritis does not have an antibiotic-specific treatment plan. The recommended antibiotics and doses differ [5]. Doxycycline 100 mg BD for 14 days is the recommended first-line regimen. Ciprofloxacin and metronidazole 500 mg OD for two weeks, or ofloxacin 400 mg OD for two weeks and then metronidazole 500 mg OD for two weeks, are used as second-line therapies [5]. If microorganisms were identified, antibiotic guidelines could be modified to address the pathogen discovered as well as any potential drug allergies the patient may have [31].

Relying on their microbiologic profiles, Cicinelli et al. documented the prescriptions of specific antibiotic regimens to infertile patients with chronic endometritis. Ciprofloxacin 500 mg twice daily for 10 days and the amoxicillin–clavulanic acid combination 2 g once daily for 8 days were administered to patients with Gram-negative and Gram-positive bacteria. The patients with mycoplasma or ureaplasma infections received 2 g of josamycin every day for 12 days, while resistant cases received 200 mg of minocycline every day for 12 days (Table 2). Even after three courses of oral antibiotic therapy, 25% of patients still had chronic endometritis, which suggests that oral antibiotic therapy for chronic endometritis is remarkably effective [31]. Patients who responded to oral antibiotic treatment had a significantly higher clinical pregnancy rate and eventual live birth rate in IVF patients (65% and 60.8%, respectively) than patients who had persistent chronic endometritis (33% and 13.3%, respectively) [31]. A correlation between the use of antibiotics and the success of IVF in patients with chronic endometritis has only been briefly described by a few authors. In order to determine the effects of antibiotic therapy for chronic endometritis on the success of IVF in patients with recurrent implantation failure, Vitagliano et al. performed a meta-analysis. Compared to patients with persistent endometrial infection, patients with cured chronic endometritis had higher rates of clinical pregnancy (OR, 4.02), live births (OR, 6.81), and implantation (OR, 3.24) [5].

It was first shown by Cicinelli et al. in 2005 that CE was accompanied by tiny mucosal proliferations (1 mm in diameter) that resembled endometrial polyps (EPs) at hysteroscopy and were referred to by the authors as “micro-polyps” [20,21]. Several studies [15,36] later confirmed this observation. The hyperplastic overgrowths of glands and stroma around a vascular core give rise to EPs, which are localized, sessile, or pedunculated projections of endometrial mucosa [34,35]. Both reproductive-aged and post-menopausal women frequently exhibit EPs. Although chronic endometrial inflammation has been definitively associated with the development of micro-polyps, it is still unknown how EPs (also known as “macro-polyps”) and CE are related. However, whether endometrial polyps or CE develop first is a question of whether the chicken or the egg came first. Endometrial polyps are strongly associated with CE. In a cross-sectional study by Kuroda et al. of 267 infertile women who underwent a hysteroscopic polypectomy for endometrial polyps, it was proven that the majority of patients had CD138-positive cells in both their endometrium and endometrial polyp samples, which were taken separately, but the number of CD138-positive cells in the polyps was noticeably higher than in the endometrium [37].

Consequently, Kuroda et al. was the first to report on the efficacy of hysteroscopic polypectomy on CE with endometrial polyps. In particular, 88.8% of women who underwent hysteroscopic polypectomy for CE with endometrial polyps were successfully treated without the use of doxycycline. The efficacy of hysteroscopic polypectomy is due to the fact that chronic endometritis includes non-infectious endometritis. It is possible that many infertile women with endometrial polyps have non-infectious CE. As a result, the majority of patients who underwent hysteroscopic polypectomy and were not administrated doxycycline were cured of CE [37].

However, hysteroscopic polypectomy is also beneficial in cases of infectious CE with endometrial polyps. Early rehabilitation from an infectious disease is encouraged by eliminating the source of infected tissues. The removal of endometrial polyps via hysteroscopic surgery can therefore be effective in treating infectious CE [37]. Notably, hysteroscopic polypectomy significantly raises the pregnancy rate. A case-control study by Rackow et al. assessed the impact of hysteroscopically detected endometrial polyps on endometrium through the use of HOXA10 and HOXA11, both established molecular markers of endometrial receptivity. Endometrial polyp-affected uteri showed a substantial decline in HOXA10 and HOXA11 messenger RNA levels compared to the controls, which may indicate decreased endometrial receptivity and impede implantation. Endometrial HOX gene expression may change in the presence of a polyp, and modifications to endometrial signaling pathways may affect endometrial receptivity. These results provide molecular evidence that confirms some clinical findings that hysteroscopic polypectomy improves pregnancy rates [38].

Ben Nagi et al. investigated the effect of hysteroscopic polypectomy on the concentration of endometrial implantation factors in uterine flushing. According to the results of this study, endometrial polyps within the uterus cavity may actually alter the amount of endometrial protein present in the uterine flushing. In comparison to the preoperative data, there was a considerable rise in the concentrations of IGFBP-1, TNFa, and osteopontin in the uterine flushing taken in the middle of the luteal phase after polypectomy [39]. Specifically, the pre- and post-polypectomy groups had significantly different IGFBP-1 concentrations, with the IGFBP-1 levels found to be lower in the women with endometrial polyps than in the women without polyps. It has been demonstrated that IGFBP-1 acts as a maternal “restraint” on trophoblast invasion by preventing their invasion into decidualized endometrial stromal cultures. Thus, in women with endometrial polypoid lesions, low levels of IGFBP-1 may cause insufficient endometrial decidualization and failure of implantation [39].

TNFa levels were noticeably elevated after surgical polypectomy. Following the excision of polyps, TNFa secretion also increased throughout the whole menstrual cycle, peaking in the secretory phase. Since TNFa is a multifunctional cytokine, its complicated action on the endometrium and the preimplantation embryo, which results in a successful implantation, is suggested by its expression at various menstrual cycle phases [39].

Osteopontin is involved in the last stages of implantation as well as the attachment of a trophoblast to the endometrial epithelium. The pre-polypectomy group’s lower concentrations and lack of a rise in the secretory phase indicate that endometrial polyps have a detrimental impact on osteopontin secretion, which may prevent the pre-implanting blastocyst from adhering to the decidua in these women [39].

The hysteroscopic polypectomy may optimize the local intrauterine environment from CE, due to the suppression of inflammatory cytokines and increase in decidual markers, both improving fertility [37]. In terms of fertility, hysteroscopic polypectomy appears to be superior to indiscriminate antibiotic use which increases the possibility of developing antibiotic resistant bacteria while suppressing the endometrial *Lactobacillus* ratio [37].

The detection rate of *Lactobacillus* was reduced in the antibiotic group compared to the non-antibiotic group, but this difference was not statistically significant, based on the most recent study of Kuroda et al., which investigated the improvement in CE and pregnancy outcomes after polypectomy with or without antibiotic therapy. Doxycycline may inhibit the endometrial Lactobacillus ratio, delaying CE healing and lowering pregnancy rates after polypectomy [37].

Τhe presence of a non-*Lactobacillus*-dominated microbiota in a receptive endometrium was associated by Moreno et al. with significant reductions in implantation, although the endometrial microbiota was not hormonally regulated during endometrial receptivity formation [40]. Based on a previous study by Ravel et al., in healthy, reproductive-aged women, the vaginal microbiome occupies states dominated by *Lactobacillus iners* and *Lactobacillus crispatus*, specifically in combination with low vaginal pH, as a result of lactic acid produced by *Lactobacillus* [41].

Thus, Kuroda et al. assessed that the endometrial polyps in CE could be treated with hysteroscopic polypectomy and unnecessary antibiotic administration should be avoided as it appears to have negative effects on pregnancy rates [37].

## 4. Conclusions

Several studies conducted thus far have confirmed that CE is linked to poor reproductive outcomes in infertile women due to the potential disturbance within the local microenvironment for the migration and embedding of the embryos, leading to implantation failure and pregnancy loss. Although there are still numerous unsolved questions on CE, the establishment of hysteroscopy as part of the diagnostic procedure seems to be important since it allows for a direct visualization of the inflammation within the cavity and consequently a more targeted biopsy. It also aids in establishing standardized diagnostic criteria merging histopathological and molecular microbiologic characteristics of CE. As far as the therapeutic use of hysteroscopy is concerned, hysteroscopic polypectomy seems to have significant fertility benefits with or without antibiotic therapy, although there is a lack of well-designed prospective studies and further studies need to be prepared in order to corroborate this finding.

## Figures and Tables

**Table 1:** Diagnostic Methods for Chronic Endometritis.

Diagnostic Method	Key Information	Reference
Prevalence of Chronic Endometritis (CE)	Ranges from 0.2% to 46%	[5]
Pathogenesis of CE	Associated with changes in the endometrial microbiome and abnormal proliferation of bacteria	[13]
Clinical Manifestations	Often asymptomatic; pelvic pain, vaginal discharge, and abnormal bleeding in some cases	[14]
Diagnosis Methods	Histopathologic examination, endometrial biopsy, hysteroscopy, and immunohistochemistry (CD138)	[15]
Hysteroscopic Findings	Micro-polyps, mucosal edema, hyperemia, and “strawberry aspect” observed	[16]
Diagnostic Agreement (2019)	Criteria include strawberry appearance, focal hyperemia, hemorrhagic patches, micro-polyps, and stromal edema	[15,22]
Hysteroscopic Scoring System (Liu et al.)	Developed with factors like endometrial hyperemia, hemorrhagic spots, and micro-polyps	[17]
Drawback of Hysteroscopy	Subjective visual evaluation, influenced by physician experience	[22]

**Table 2:** Recommended Antibiotic Therapies for Chronic Endometritis.

Antibiotic	Dosage	Duration	Microorganisms Targeted	Comments	Reference
Doxycycline	100 mg BD	14 days	Broad-spectrum, Gram-negative, Gram-positive	First-line regimen	[5]
Ciprofloxacin + Metronidazole	500 mg OD + 500 mg OD	14 days		Second-Line	[5]
Ofloxacin + Metronidazole	400 mg OD + 500 mg OD	14 days		Second-Line	[5]
Ciprofloxacin	500 mg BD	10 days	Gram-negative bacteria	Second-Line	[31]
Ciprofloxacin + Amoxicillin-clavulanic acid	500 mg BD + 2 g OD	8 days	Gram-negative and Gram-positive bacteria	Specific Regimen	[31]
Josamycin	2 g OD	12 days	Mycoplasma or Ureaplasma	Specific infections	[31]
Josamycin + minocycline	2 g OD + 200 mg OD	12 days	Mycoplasma or Ureaplasma (resistant cases)	Specific infections	[31]

## Data Availability

Data used in this study are presented within the manuscript.
